# Schottky junction/ohmic contact behavior of a nanoporous TiO_2_ thin film photoanode in contact with redox electrolyte solutions

**DOI:** 10.3762/bjnano.2.15

**Published:** 2011-02-28

**Authors:** Masao Kaneko, Hirohito Ueno, Junichi Nemoto

**Affiliations:** 1 The Institute of Biophotochemonics Co.Ltd., 2-1-1 Bunkyo, Mito, 310-8512 Japan

**Keywords:** cyclic voltammogram of titanium dioxide photoanode, flat band potential, nanoporous TiO_2_ thin film, photocurrent, Schottky junction and ohmic contact

## Abstract

The nature and photoelectrochemical reactivity of nanoporous semiconductor electrodes have attracted a great deal of attention. Nanostructured materials have promising capabilities applicable for the construction of various photonic and electronic devices. In this paper, a mesoporous TiO_2_ thin film photoanode was soaked in an aqueous methanol solution using an O_2_-reducing Pt-based cathode in contact with atmospheric air on the back side. It was shown from distinct photocurrents in the cyclic voltammogram (CV) that the nanosurface of the mesoporous n-TiO_2_ film forms a Schottky junction with water containing a strong electron donor such as methanol. Formation of a Schottky junction (liquid junction) was also proved by Mott–Schottky plots at the mesoporous TiO_2_ thin film photoanode, and the thickness of the space charge layer was estimated to be very thin, i.e., only 3.1 nm at −0.1 V vs Ag/AgCl. On the other hand, the presence of [Fe(CN)_6_]^4−^ and the absence of methanol brought about ohmic contact behavior on the TiO_2_ film and exhibited reversible redox waves in the dark due to the [Fe(CN)_6_]^4−/3−^ couple. Further studies showed that multiple Schottky junctions/ohmic contact behavior inducing simultaneously both photocurrent and overlapped reversible redox waves was found in the CV of a nanoporous TiO_2_ photoanode soaked in an aqueous redox electrolyte solution containing methanol and [Fe(CN)_6_]^4−^. That is, the TiO_2_ nanosurface responds to [Fe(CN)_6_]^4−^ to give ohmic redox waves overlapped simultaneously with photocurrents due to the Schottky junction. Additionally, a second step photocurrent generation was observed in the presence of both MeOH and [Fe(CN)_6_]^4−^ around the redox potential of the iron complex. It was suggested that the iron complex forms a second Schottky junction for which the flat band potential (*E*_fb_) lies near the redox potential of the iron complex.

## Introduction

Photoelectrocatalytic reactions at semiconductor electrodes were investigated before the 1960s [1–2]. A semiconductor electrode forms a type of Schottky junction with liquid electrolytes called a liquid junction, which generates a photocurrent. A crystalline n-TiO_2_ photoanode to decompose water by UV light attracted a great attention [3]; organic compounds have also been similarly decomposed [4–5]. Later, a nanoporous TiO_2_ thin film was applied to a dye-sensitized solar cell (DSSC) [6] in which the nanoporous TiO_2_ film works in an organic liquid electrolyte solution as an electron acceptor and conductor rather than as a liquid junction semiconductor. With regard to this invention, the photoreactivity of nanoporous semiconductors has been an important issue in potential applications. There has been much argument as to the nature of the interface between nanoporous semiconductor and an aqueous redox electrolyte solution such as K_4_[Fe(CN)_6_] [7–10]. The nature of such an interface should strongly depend on the type and concentration of the redox substrate present in the solution phase due to the nanostructure. It was reported that cathodic photocurrents were obtained at a nanocrystalline TiO_2_ film electrode due to oxygen reduction in alkaline solutions [7]. Visible light sensitization of TiO_2_ by surface complexation with [Fe(CN)_6_]^4−^ has also been reported [8]. In such a case it was suggested that the electron injection occurs at only one or a few Ti centers located very close to the adsorbed location of the iron cyanide complex [9]. As one of the applications in solid-state electronics, an optoelectronic logic device was fabricated from nanocrystalline TiO_2_ modified with hexacyanoferrate anions [10].

The present authors have reported a cell composed of a nanoporous semiconductor photoanode and an O_2_-reducing cathode that can efficiently photodecompose various bio-related compounds in water [11–12]. When ammonia was present in water in contact with a nanoporous TiO_2_ photoanode, the semiconductor formed a kind of Schottky junction, which induced efficient decomposition of ammonia with a high internal quantum efficiency (i.e., the number of decomposed molecules per photon activating an NH_3_ molecule) of over 100 (=10^4^%) through auto-oxidative decomposition of the activated ammonia [12]. In subsequent research we found that a mesoporous TiO_2_ semiconductor film forms both a Schottky junction as well as exhibiting partial ohmic contact behavior in a redox electrolyte solution and this multi-nature behavior is reported in this publication.

## Results and Discussion

**Figure 1:**
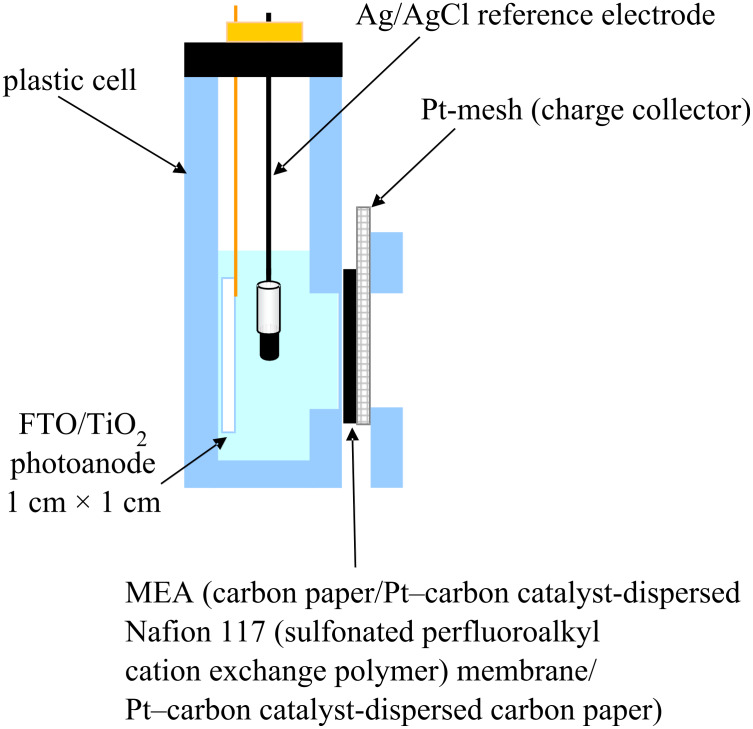
Side view of a photoelectrochemical cell (1 cm × 1 cm × 3 cm) used for CV measurements with membrane electrode assembly (MEA) attached to a Pt-mesh cathode.

**Figure 2:**
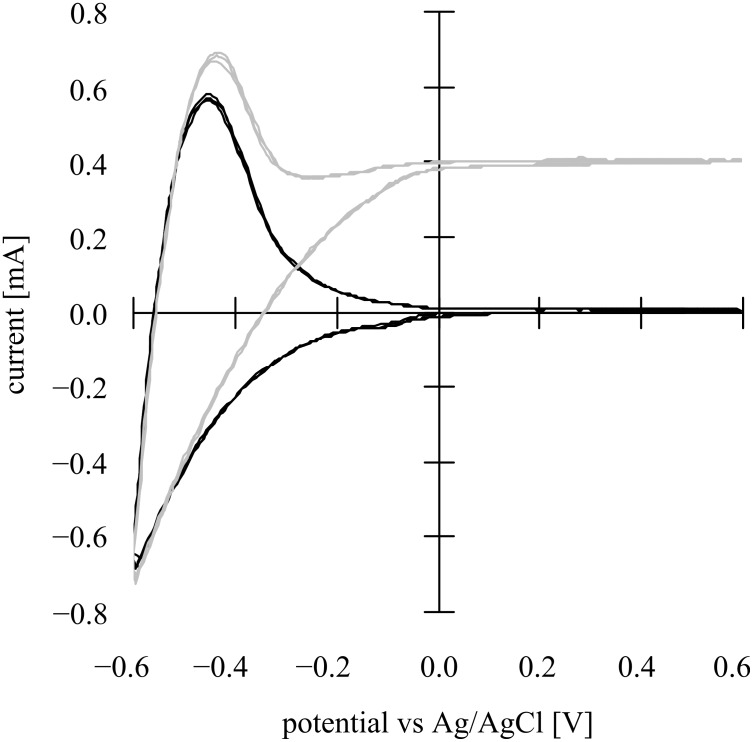
Cyclic voltammograms both in the dark (black line) and with light irradiation (gray line) at a nanoporous TiO_2_ photoanode (1cm x 1cm) in an aqueous solution of 10 wt % methanol (+ 0.1 M Na_2_SO_4_, pH 8.5) under an Ar atmosphere with MEA-attached Pt (1 cm × 1 cm) as the counter electrode and Ag/AgCl as the reference electrode. The light intensity was 3.5 mW·cm^−2^ (UV-A region). Scan rate: 100 mV·s^−1^.

The cyclic voltammogram (CV) at a nanoporous TiO_2_ (T/SP, Solaronix) thin film photoanode soaked in a 10 wt % aqueous methanol solution (+ 0.1 M Na_2_SO_4_ electrolyte, pH 8.5) in a cell (Figure 1) under an Ar atmosphere is shown in Figure 2.

In the dark in the cathodic scan from 0.6 V to −0.6 V vs Ag/AgCl, the cathodic current starts to increase around 0 V vs Ag/AgCl most probably due to O_2_ reduction in the first instance and then H^+^ reduction, and in the reverse scan re-oxidation of the generated H_2_ occurs whose peak is located around −0.45 V vs Ag/AgCl. By contrast, under irradiation the CV shows distinct photocurrents that reach a plateau around 0 V, demonstrating clearly that band bending occurs at the TiO_2_/liquid interface due to a kind of Schottky junction formation as noted below.

**Figure 3:**
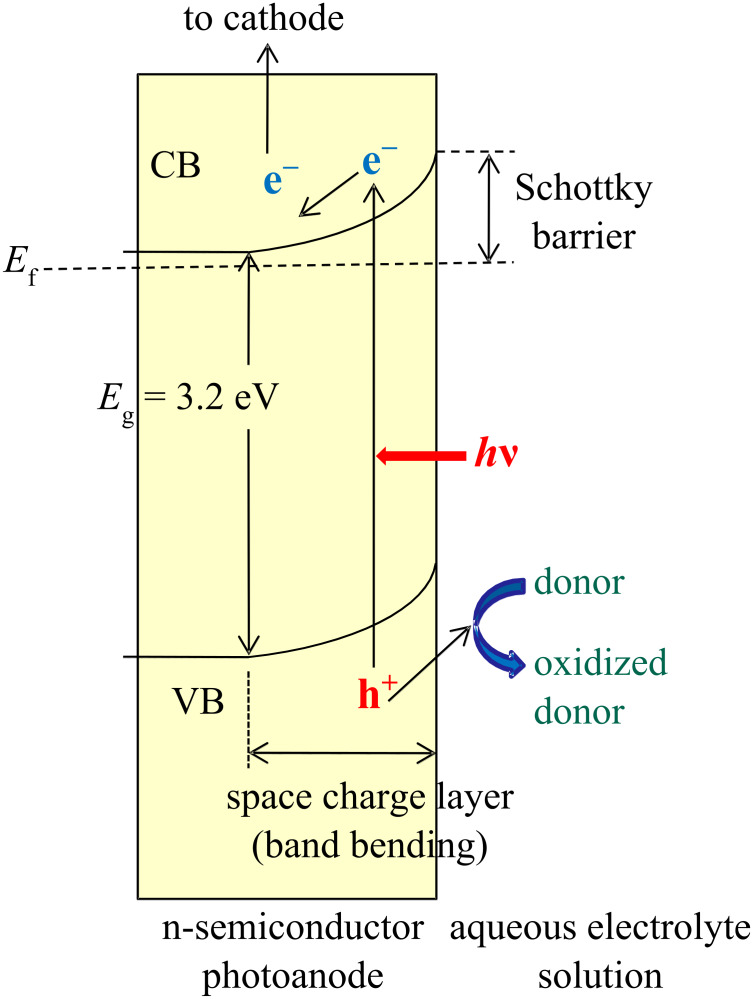
Formation of a Schottky barrier (junction) in an n-semiconductor photoanode at the interface with aqueous electron donor/electrolyte solution. *E*_f_ = Fermi level, *E*_g_ = band gap, VB = valence band, CB = conduction band.

Since much argument exists as to the nature of the contact between a mesoporous TiO_2_ thin film and an electrolyte solution, this will first be discussed here. Taking an n-type semiconductor (SC), such as TiO_2_ and a metal as an example, we adopt the case that the Fermi level (*E*_f_) of the SC is higher (i. e., more negative) than that of the metal. For an n-SC, the Fermi level is located at a slightly lower (more positive) level than the lower edge of the conduction band (CB) (Figure 3). When the n-SC is in contact with the metal (or the same with an electrolyte solution instead of the metal), the electrons in the SC are transported to the vacant level of the metal (or electrolyte solution), which makes the SC positively charged, and the metal (or electrolyte solution) negatively charged. As the result the band structure of the SC (both the valence band (VB) and the conduction band) is bent as shown in Figure 3.

This curved portion of the band structure is called a space charge layer (also called a depletion layer, where electrons are depleted). Under this condition, on irradiation by photons at the interface between the SC and the metal (or electrolyte solution), the energy of which is larger than the bandgap (*E*_g_) of the semiconductor (for TiO_2_ *E*_g_ ~ 3.2 eV, wavelength < 390 nm), excitation of electrons from the VB to CB in the SC takes place leaving holes in the VB. The electron and the hole form an exciton (excited electron–hole pair), which is usually short-lived and recombines if there is no driving force to separate them. However, when the band structure is bent as in Figure 3 for an n-SC, the hole can migrate towards the SC interface, and the electron can migrate towards inside of the semiconductor bulk, thus the hole and the electron are now separated. After such migration of holes and electrons, when an electron donor (such as ethanol) is present in the contacted liquid phase, the holes can oxidize the donor in the liquid, and the electrons are transported first to the fluorine-doped tin oxide (FTO, SnO_2_:F) conductive layer through TiO_2_ grain boundaries and then to the cathode reducing electron acceptor there (O_2_ in the present case).

In a Schottky junction, under the conditions when the band structure is flat without any bending, the Fermi level is called the flat band potential (*E*_fb_). When an n-SC (i.e., photoanode) and a cathode are soaked in an electrolyte solution where an electron donor is present, and the anodic potential is applied to the SC under irradiation, anodic photocurrents begin to be generated due to band bending when the applied potential is shifted from cathodic polarization towards the anodic direction thus preventing electron–hole recombination of the exciton. If such a space charge layer of a Schottky junction does not exist, the holes and the electrons present as an exciton formed by photoirradiation recombine quickly and preferentially without generating a photocurrent. The photocurrents shown in the CV (Figure 2) evidently proves that a Schottky junction is formed in the nanostructured TiO_2_ thin film. It is quite unambiguous from Figure 2 that, similar to conventional semiconductor electrode photoelectrochemistry, the nanostructured TiO_2_ also forms a Schottky junction in the redox electrolyte solution generating photocurrents.

For a Schottky junction semiconductor, the Mott–Schottky relation (Equation 1) is obtained [4], where *C*_sc_ is the capacitance of the space charge layer [F·m^−2^], ε the relative permittivity (ε of TiO_2_ = 85.8 and 170, anisotropic), ε_0_ the vacuum permittivity (8.854 × 10^−12^ F·m^−1^), *q* the elementary electric charge (1.602 × 10^−19^ C), *N* the carrier density [m^−3^], *E* the applied potential [V], *E*_fb_ the flat band potential [V], *k*_B_ the Boltzman constant (1.380 × 10^−23^ J·K^−1^), and *T* the absolute temperature [K].

[1]
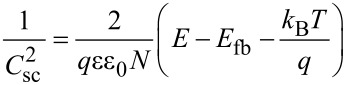


When *d* [m] is the thickness of the space charge layer, *C*_sc_ can be approximated to (ε·ε_0_)/*d*, so that *d* is estimated by Equation 2.

[2]
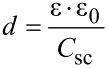


When measuring the capacitance of the SC electrode, if plots of 1/*C*_sc_^2^ against the applied potential (*E*) exhibit a linear relationship, the formation of a Schottky barrier is proved. From the intercept (= *E* − *E_fb_* – (*k*_B_* T*)*/q*) of the plots on the potential axis, the flat band potential is obtained, and from the slope (= 2/(ε·ε_0_·*q*·*N)*), the carrier density *N* can be estimated.

**Figure 4:**
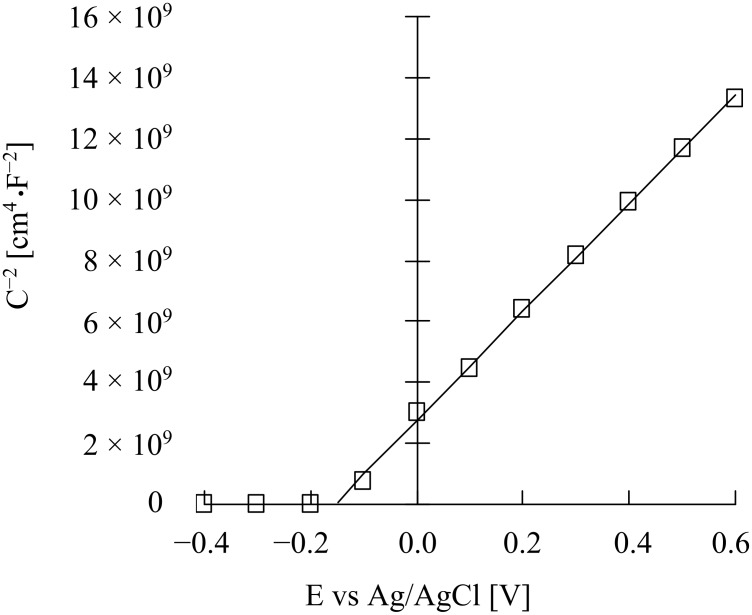
Mott–Schottky plot of a nanoporous TiO_2_ thin film coated on FTO in contact with a 10% aqueous methanol solution (+ 0.1 M Na_2_SO_4_) under irradiation. Measured with 100 Hz frequency and AC amplitude = 10 mV.

The flat band potential *E*_fb_ of a nanoporous TiO_2_ thin film photoanode soaked in water was measured by Mott–Schottky plots in a 10 wt % MeOH aqueous solution containing 0.1 M Na_2_SO_4_ (pH 8.5) under irradiation, and the results are shown in Figure 4.

Since the plot exhibits a linear relationship this proves that the TiO_2_ film forms a Schottky junction. From Equation 1 and Equation 2, the flat band potential *E*_fb_, carrier density *N*, and the thickness of space charge layer *d*_sc_ were calculated and are shown in Table 1. For the calculation, since the relative permittivity ε of TiO_2_ is anisotropic (85.8 and 170), we used both the values in the calculation, and thereafter took their average values considering the macroscopically amorphous nature of the mesoporous TiO_2_ film. Since the space charge layer thickness (*d*_sc_) is dependent on the applied potential, we calculated the value at the applied potential of −0.1 V vs Ag/AgCl at which the CV under irradiation approaches a saturated value as shown in the Figure 2.

**Table 1:** Flat band potential *E*_fb_, space charge layer thickness *d*_sc_ of the Schottky junction, and carrier density *N* in a mesoporous TiO_2_ thin film coated on FTO obtained by the Mott–Schottky plots shown in Figure 4. *d*_sc_ and *N* are average values when ε of TiO_2_ = 85.8 and 170.

*E*_fb_ vs Ag/AgCl at pH 8.5 [V]	−0.16
*d*_sc_ at −0.1 V vs Ag/AgCl [nm]	3.10
*N* [cm^−3^]	6.96 × 10^19^

**Figure 5:**
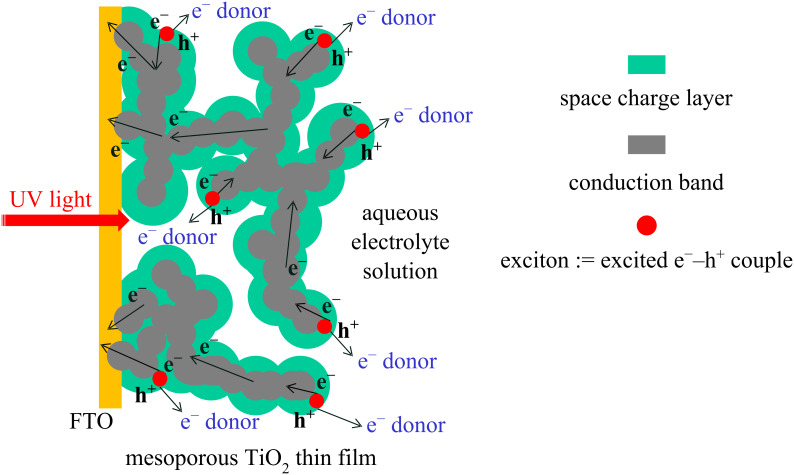
Schematic representation for the formation of continuous Schottky junctions with space charge layer (i.e., depletion layer, green region) and conduction band (CB, gray region) in a nanoporous n-TiO_2_ thin film formed at the interface with an aqueous electrolyte solution containing an electron donor. In the space charge layer holes (h^+^) of the excitons (excited e^−^–h^+^ couples, red circle) migrate in the VB onto the TiO_2_ surface reacting with the e^−^ donor, and electrons (e^−^) of the exciton migrate into the bulk CB where e^−^ then migrate through TiO_2_ grain boundaries and finally into the conductive layer on FTO. It should be noted that the 3-D structured nano-ordered thin film is shown as a 2-D picture here.

The obtained flat band potential was −0.16 V vs Ag/AgCl at pH 8.5 under irradiation. The space charge layer thickness was only 3.10 nm (at −0.1 V vs Ag/AgCl), much thinner than that of conventional SC electrodes for which it is 100 nm to 1 mm. This very thin space charge layer is reasonable since the present mesoporous TiO_2_ thin film consists of small particles of on average 13 nm diameter. The band structure of the nanostructured TiO_2_ film can therefore be depicted as in Figure 5. It should be noted here that the 3-D structured thin film is shown as a 2-D picture. The thin space charge layer is located at the interface between TiO_2_ and the liquid, and the band structure (CB and VB) in the TiO_2_ bulk is interconnected through the TiO_2_ grain boundaries forming continuous CB electron-transporting channels from the space charge layer to reach the conductive layer on the FTO. When TiO_2_ is excited by UV light, excitons are formed, but many of these would recombine simply wasting the excitation energy if band bending did not exist. However, due to the band bending in the space charge layer formed at the TiO_2_/liquid interface, the excitons formed here would be separated into electrons and holes due to the slope of the VB and CB bands, the h^+^ then being reduced by MeOH present in the liquid, and the e^−^ being transported in the CB through TiO_2_ grain boundaries to the counter cathode via the FTO. As reported earlier by us [13], resistances at the grain boundaries are low for such mesoporous TiO_2_ thin film as investigated by an alternating current impedance spectroscopy.

The electron conductivity of TiO_2_ itself is not high. However, the carrier density *N* of 6.96 × 10^19^ cm^−3^ obtained from Figure 4 was high, in the order as that of graphite, indicating that the mesoporous TiO_2_ thin film can function as a good electron-conductive material under irradiation conditions when a strong electron donor is present in the liquid. It should be noted that in a dye-sensitized solar cell (DSSC) [6], a mesoporous TiO_2_ thin film also functions as an electron conductor under irradiation due to the large electron density injected from the photoexcited state of TiO_2_-attached dye molecules.

**Figure 6:**
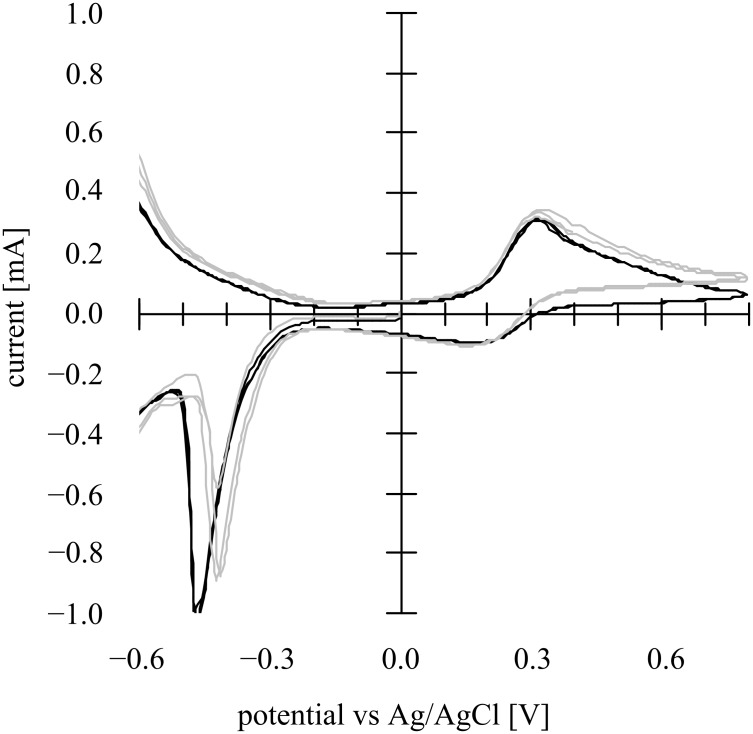
Cyclic voltammogram in the dark (black line) and with light irradiation (gray line) at a nanoporous TiO_2_ photoanode (1 cm × 1 cm) in a 400 μM [Fe(CN)_6_]^4−^ aqueous solution (+ 0.1 M Na_2_SO_4_, pH 8.5) under an Ar atmosphere with MEA (1 cm × 1 cm) as the counter electrode and Ag/AgCl as the reference electrode. Light intensity 3.5 mW·cm^−2^ (UV-A region). Scan rate: 100 mV·s^−1^.

The CV of the nanoporous TiO_2_ (T/SP, Solaronix) thin film photoanode soaked in an aqueous solution containing only 400 μM [Fe(CN)_6_]^4−^ (+ 0.1 M Na_2_SO_4_, pH 8.5) without methanol is shown in Figure 6.

In the dark, the CV showed clear reversible redox waves typical for the [Fe(CN)_6_]^4−/3−^ couple at 0.26 V vs Ag/AgCl at pH 8.5, demonstrating that the nanoporous TiO_2_ film shows ohmic contact behavior with the [Fe(CN)_6_]^4−^ complex. This would be due to the thin space charge layer structure; the applied positive charges can oxidize the Fe(II) complex through the space charge layer, and then re-reduce the oxidized Fe(III) complex in the reverse scan. Under irradiation, only a low photocurrent was observed due to the absence of MeOH since the electron donating ability of the Fe complex is low. It should be noted here that the redox potential of the iron complex obtained was much more positive than that of this complex (0.69 V vs SHE at pH 0, corresponding to −0.03 V vs Ag/AgCl at pH 8.5), which could be interpreted by the redox potential shift of the iron complex attached to the dissociated surface structure by ligand exchange of the Fe^2+/3+^ center between CN^−^ and Ti–O^−^.

**Figure 7:**
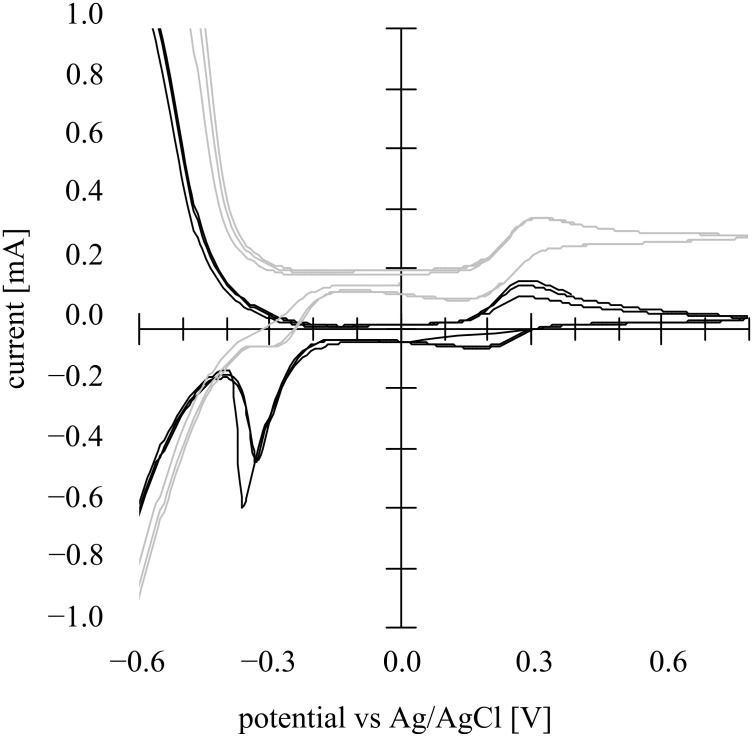
Cyclic voltammogram in the dark (black line) and light irradiation (gray line) at a nanoporous TiO_2_ photoanode (1 cm × 1 cm) in a 10 wt % aqueous methanol solution containing 400 μM [Fe(CN)_6_]^4−^ (+ 0.1 M Na_2_SO_4_, pH 8.5) under an Ar atmosphere with MEA (1 cm × 1 cm) as the counter electrode and Ag/AgCl as the reference electrode. Light intensity 3.5 mW·cm^−2^ (UV-A region). Scan rate: 100 mV·s^−1^.

When both the methanol and the [Fe(CN)_6_]^4−^ were present in the aqueous phase, the behavior was interesting. In the dark, the CV also showed clear reversible redox waves for the [Fe(CN)_6_]^4−/3−^ couple (Figure 7) at 0.26 V vs Ag/AgCl (pH 8.5).

Under irradiation conditions an anodic photocurrent was clearly observed in the CV curve due to the electron donating MeOH, and, in addition, the photocurrent CV curve was overlapped by the reversible redox waves of the iron complex, showing that the nanoporous TiO_2_ film can have simultaneously both Schottky junction and ohmic contact behavior. Sensitization and electron transfer in TiO_2_ nanoparticles and nanoporous electrodes by [Fe(CN)_6_]^4−^ has been thoroughly investigated [8–10], but such multi-nature behavior of both Schottky junction and ohmic contact has not been reported.

**Figure 8:**
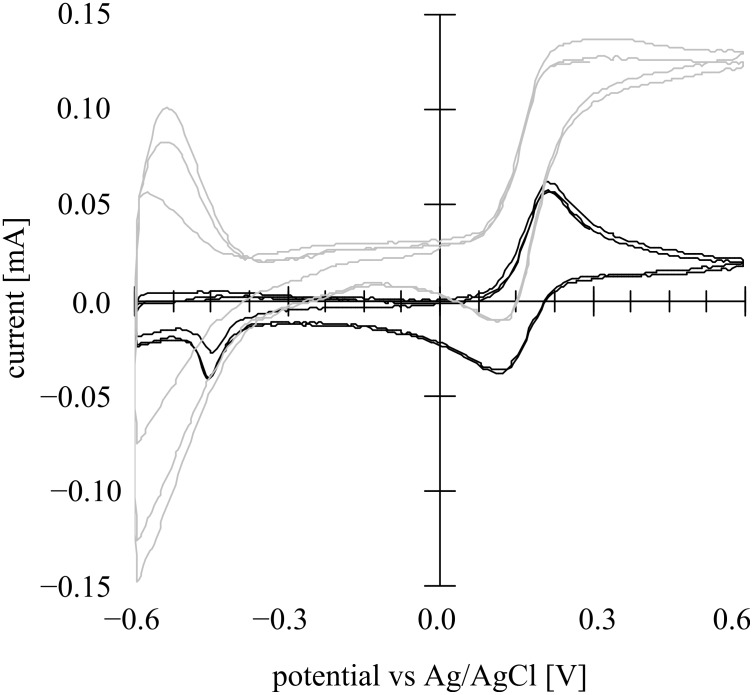
Cyclic voltammogram in the dark (black line) and on irradiation (gray line) at a nanoporous TiO_2_ (G2) photoanode (1 cm × 1 cm) composed of larger particles (500 nm) soaked in a 10 wt % aqueous methanol solution containing 400 μM [Fe(CN)_6_]^4−^ (+ 0.1 M Na_2_SO_4_, pH 8.5) under an Ar atmosphere using a 10 mL cylindrical cell with a Pt black coated Pt plate cathode as the counter electrode and Ag/AgCl as the reference electrode. Light intensity 3.5 mW·cm^−2^ (UV-A region). Scan rate: 100 mV·s^−1^.

In order to investigate further the behavior in Figure 7, larger size (500 nm) TiO_2_ (G2, rutile >95%, note that anatase-rich sample is not available and difficult to prepare for this particle size) was used instead of the Ti-nanoxide T/SP (average diameter 13 nm, anatase >90%), and the CVs at the mesoporous G2 thin film-coated photoanode in the presence of both methanol and [Fe(CN)_6_]^4−^ in the aqueous phase are shown in Figure 8 in the dark and under irradiation. For this measurement a 10 mL cylindrical cell was used with a Pt black coated Pt plate cathode. It should be noted that, in the anodic direction CV curve under irradiation, a second photocurrent generation was observed starting from 0.1 V vs Ag/AgCl accompanying the oxidation of the iron complex. The interpretation might not be simple, but this might suggest that the iron complex, most probably attached to the TiO_2_ surface, induced a second band structure whose *E*_fb_ lies near the redox potential of the iron complex on the TiO_2_ surface.

**Figure 9:**
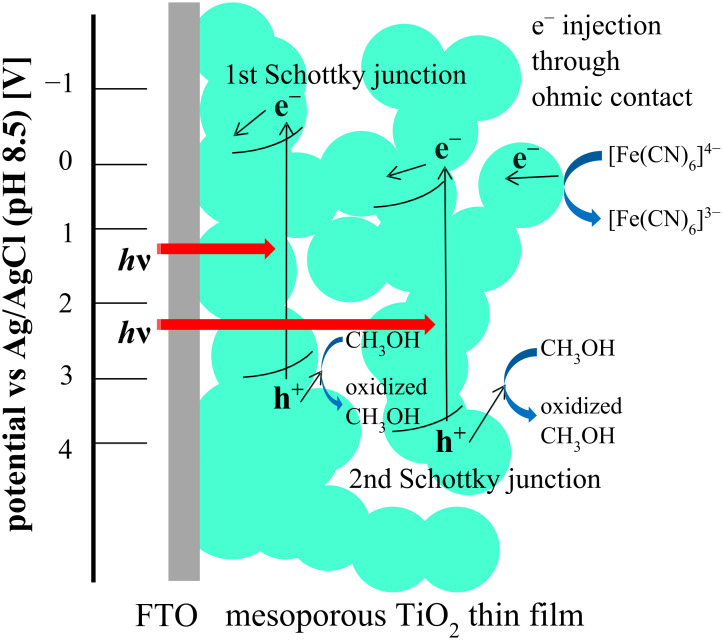
Schematic representation of a two-step Schottky junction/ohmic contact behavior of a nanoporous n-TiO_2_ thin film.

The photocurrents increased with repeated scans. In a previous paper [14] by one of the present authors (MK), both Schottky junction and ohmic contact behavior were found at a single crystal CdS photoanode with RuO_2_ fine powder attached to the surface and coated with a thin film of polymer-pendant Ru(bpy)_3_^2+^ on top of the RuO_2_. It was clear that the CdS formed a Schottky junction with the redox electrolyte solution, and in addition, the RuO_2_ formed an ohmic contact with the CdS, and the coated cationic Ru(bpy)_3_^2+^ polymer electrostatically incorporating the anionic K_4_[Fe(CN)_6_] exhibited photocurrents that overlapped redox waves of the iron complex. While in the multiple Schottky junctions/ohmic behavior of the nanoporous TiO_2_ soaked in a methanol aqueous solution containing [Fe(CN)_6_]^4−^ reported in the present paper, a second photocurrent was observed around the redox potential of the iron complex. This Schottky junction/ohmic contact behavior could schematically be represented by Figure 9. Such multiple Schottky junctions/ohmic contact behavior must originate from the nanostructured nature of the nanoporous TiO_2_ thin film photoanode.

## Conclusion

The nanosurface of a mesoporous n-TiO_2_ film forms, in principle, a Schottky junction with an aqueous electrolyte solution, so that, in the presence of strong electron-donating compound (MeOH in the present case), clear photocurrents were generated (Figure 2). From the Mott–Schottky plots, the Schottky junction formation was proved, and the space charge layer thickness was estimated to be 3.1 nm at the applied potential of −0.1 V vs Ag/AgCl. The TiO_2_ nanosurface itself also forms ohmic contact with [Fe(CN)_6_]^4−^ complex giving ohmic redox waves (Figure 5). The presence of both MeOH and the iron complex induced simultaneous Schottky junction and ohmic contact behavior exhibiting photocurrents overlapped with the redox waves of the iron complex (Figure 7). In addition, it was suggested that the iron complex formed a second Schottky junction on the semiconductor surface for which the *E*_fb_ lies at the redox potential of the iron complex (Figure 8). This Schottky junction/ohmic contact behavior is schematically shown in Figure 9. The nature of a nanoporous semiconductor film soaked in liquid could be tuned easily by the presence of various electron donors and acceptors and other redox compounds in the liquid phase. Such Schottky junction/ohmic contact characteristics of nanoporous semiconductor thin films could be applied to a variety of photonic and electronic devices in the future.

## Experimental

### Materials and electrodes preparation

To prepare a nanoporous TiO_2_ film, Ti-nanoxide paste (T/SP, average particle size 13 nm, anatase >90%) was purchased from Solaronix SA, Aubonne, Switzerland. Larger size TiO_2_ powders, G2 (500 nm, rutile >95%) was purchased from Showa Denko Co., Ltd, Japan. F-doped SnO_2_ conductive glass (FTO, surface resistance 10 Ω·cm^−2^) was purchased from AGC Fabritec Co., Ltd., Japan. All the other chemicals were of the purest grade commercially available and used as received. Ti-nanoxide (T/SP) paste or G2 paste prepared by the reported procedure [11] was coated on an FTO (2 cm × 1 cm) by a squeeze coating method with a coated area of 1 cm × 1 cm. For this procedure, adhesive tape (thickness about 70 μm) was used as a spacer to adjust the TiO_2_ film thickness to around 10 μm after calcination. The TiO_2_ paste film on FTO was dried at room temperature, and then calcined at 450 °C for 30 min to give a nanoporous thin film of about 10 μm thickness with a roughness factor of about 1000. For Figure 2 and Figure 5–7, T/SP TiO_2_ was used and, in order to bring the other side of a Pt cathode in contact with air, a membrane electrode assembly (MEA) (1 cm × 1 cm) purchased from FC Development Co., Ltd., Japan was used. This is composed of three layers, i.e., [carbon paper/Pt–carbon catalyst-dispersed Nafion 117 (sulfonated perfluoroalkyl cation exchange polymer) membrane/Pt–carbon catalyst-dispersed carbon paper], for which the first layer is in contact with the electrolytes liquid, and the last layer is exposed to ambient air (Figure 1). For Figure 8, the larger size G2 TiO_2_ was used, and Pt black was deposited electrochemically from K_2_[PtCl_6_] on a Pt plate (1 cm × 1 cm) in order to use it as an O_2_-reduction cathode in the liquid phase.

### Cell, irradiation, and measurements

A cell (1 cm × 1 cm × 3 cm) was designed and fabricated by plastic plates as shown in Figure 1. An aqueous solution of 10 wt % methanol containing either 0 or 400 μM potassium hexacyanoferrate(II), K_4_[Fe(CN)_6_], as the redox electrolyte was used which also contained 0.1 M Na_2_SO_4_ at pH 8.5. A nanoporous TiO_2_ thin film photoanode and a Ag/AgCl reference electrode were soaked in the aqueous redox electrolyte solution, and the MEA O_2_-reducing cathode mentioned in the last section was used as shown in Figure 1 so that one side is in contact with the liquid phase and the other side in contact with atmospheric air. Ar gas was bubbled into the solution to displace the air by Ar. For the measurement depicted in Figure 8, a cylindrical cell of 13 mL was used with a TiO_2_/FTO photoanode, Pt black coated Pt plate cathode, and an Ag/AgCl reference electrode.

Electrochemical measurements were conducted with an HZ-3000 automatic polarization system (Hokuto Denko Co., Ltd., Japan), and Mott–Schottky plots were obtained in combination with a 5020 frequency analyzer (NF Electronic Instruments, Japan) with 100 Hz frequency and AC amplitude of 10 mV. The light source was a 500 W xenon lamp adjusted to irradiate white light in the 3.5 mW·cm^−2^ UV-A region (1 sun condition). The measurements were conducted with the liquid phase under an Ar atmosphere at 25 °C for all the data. The UV-A region light (290–390 nm) intensity was measured by a UV light meter (model UV-340, CUSTOM Co., Ltd.).
